# Loss of L1CAM from gastric epithelial cells and extracellular vesicles promotes *Helicobacter pylori*-driven cancer progression and invasiveness

**DOI:** 10.1186/s12964-026-03087-5

**Published:** 2026-07-25

**Authors:** Supriya Samal, Smaran Banerjee, Abhisek Brahma, Prabin Bawali, Aranya Pal, Debashish Chakraborty, Indrajit Poirah, Gautam Nath, Sasmita Panda, Niranjan Rout, Shivaram P. Singh, Saurabh Chawla, Duane T. Smoot, Hassan Ashktorab, Asima Bhattacharyya

**Affiliations:** 1 School of Biological Sciences, National Institute of Science Education and Research (NISER) Bhubaneswar, Jatni, Odisha 752050 India; 2 Homi Bhabha National Institute, Training School Complex, Anushakti Nagar, Mumbai, 400094 India; 3 Department of Gastroenterology, Acharya Harihar Post Graduate Institute of Cancer, Cuttack, Odisha 753007 India; 4 Department of Pathology, Acharya Harihar Post Graduate Institute of Cancer, Cuttack, Odisha 753007 India; 5 Digestive Diseases Centre, Beam Diagnostics Building, Bajrakabati Road, Cuttack, Odisha 753001 India; 6 Department of Medicine, Meharry Medical Center, Nashville, TN 37208 USA; 7 Department of Medicine, Howard University, Washington DC, 20060 USA; 8 Centre for Interdisciplinary Sciences (CIS), NISER Bhubaneswar, Jatni, Odisha 752050 India

**Keywords:** ADAM10/17, Epithelial-to-mesenchymal transition, Extracellular vesicle, Gastric cancer, Gastric epithelial cells, *Helicobacter pylori*, L1CAM, Proteolysis

## Abstract

**Background:**

*Helicobacter pylori* (*Hp*) infection is a major risk factor for gastric cancer (GC). While *Hp*-driven alterations in host signaling have been widely studied, the contribution of extracellular vesicles (EVs) to this process remains largely unexplored. Cell adhesion molecules play key roles in maintaining epithelial integrity and their dysregulation is often associated with malignant transformation. L1 cell adhesion molecule (L1CAM), a transmembrane protein involved in cell-cell interactions and signaling, has been implicated in cancer progression but its regulation in the context of *Hp* infection remains unclear. Here, we aimed to determine the relevance of L1CAM regulation and its EV-associated dynamics in *Hp* infection-induced GC.

**Methods:**

L1CAM status was assessed in *Hp*-positive gastric biopsy specimens by immunofluorescence microscopy and by western blotting of *Hp*-infected gastric epithelial cells (GECs). EVs from infected GECs were isolated and characterized. Functional assays, including proliferation, migration and xenograft studies were performed using infected or uninfected *L1CAM*-overexpressing as well as empty-vector cell-derived EVs. Integrin modulation by L1CAM was assessed by immunoblotting and microscopy in in vitro studies, patient samples and xenograft models using *L1CAM*-modulated cells. Epithelial-mesenchymal transition (EMT) markers, L1CAM proteolysis and inhibition studies were analyzed by immunoblotting to delineate the underlying molecular mechanisms.

**Results:**

*Hp* infection caused a marked loss of the full-length (FL) L1CAM in GECs and secreted GEC-EVs. A similar decrease was observed in *Hp*-positive GC tissues and plasma-derived EVs. Mechanistically, *Hp*-driven loss of FL-L1CAM resulted from the metalloproteinase ADAM10-mediated cleavage and was accompanied by the appearance of L1CAM fragments in infected cells or their EVs. EVs derived from infected cells significantly enhanced proliferation and migration of non-cancerous GECs in vitro as well as promoted tumor growth in vivo. In contrast, EVs from uninfected cells attenuated these malignant traits, suggesting that *Hp*-associated proteolytic depletion of FL-L1CAM fosters a tumor-promoting phenotype. Additionally, loss of FL-L1CAM induced EMT and selectively decreased αVβ1 integrin across infected cells, patient biopsies and xenograft tissues, defining a distinct *Hp*-driven L1CAM-ADAM10-αVβ1 signaling axis.

**Conclusion:**

*Hp*-induced proteolytic cleavage of L1CAM represents a distinct molecular signature of *Hp*-associated GC. FL-L1CAM-depleted EV may serve as a promising minimally invasive biomarker in *Hp*-driven gastric carcinogenesis.

**Supplementary Information:**

The online version contains supplementary material available at https://doi.org/10.1186/s12964-026-03087-5.

## Introduction

Gastric cancer (GC) remains one of the most lethal malignancies worldwide largely due to its aggressive metastatic behavior and late detection. Chronic infection with *Helicobacter pylori* (*Hp*), which affects nearly half of the global population, is one of the strongest known risk factors for GC. Persistent *Hp* infection drives chronic gastric inflammation and rewires host signaling network through genetic and post-translational modifications, thereby promoting malignant transformation and disease progression [1, 2]. Despite advances in therapeutic strategies, overall survival of GC patients remains poor [3], underscoring the urgent need for improved early detection approaches and novel molecular targets.

Within the GC tumor microenvironment (TME), extracellular vesicles (EVs) function as critical mediators of intercellular communication. These nano-sized membranous vesicles transfer bioactive cargo between gastric epithelial cells (GECs) and surrounding stromal or immune cells, thereby shaping tumor growth, metastasis and therapy resistance [4, 5]. Emerging evidence highlights the importance of EVs in GC progression: hypoxia-induced, miR-301a-3p-enriched exosomes promote metastasis [6], GC-derived EVs carrying the E3 ubiquitin ligase TRIM3 suppress tumor growth [7] and LINC01559-loaded exosomes accelerate GC progression [8]. *Hp* infection alters the molecular composition of GEC-derived EVs [9]. However, the dynamics and functional consequences of these changes remain poorly understood.

L1 cell adhesion molecule (L1CAM/CD171) is a transmembrane glycoprotein of the immunoglobulin (Ig) superfamily, originally identified in the nervous system but aberrantly expressed in multiple cancers [10]. The functional role of L1CAM in GC remains incompletely understood, with evidence suggesting context-dependent effects [11, 12]. Structurally, L1CAM comprises an extracellular N-terminal region with six Ig domains and five fibronectin-type III (FNIII) repeats, followed by a transmembrane region and a conserved cytoplasmic tail [13]. L1CAM, through its N-terminal ectodomains, mediates homophilic interactions as well as heterophilic binding to other adhesion molecules, including integrins, thereby activating signaling pathways that regulate cell adhesion, migration and plasticity [14]. Importantly, these interactions can be disrupted by proteolytic cleavages within the ectodomain of L1CAM, generating soluble and membrane-associated fragments with distinct biological activities [15].

The role of *Hp*-infected EVs and *Hp*-specific circulating biomarkers in GC remains poorly understood. In particular, the status, regulation and functional significance of L1CAM during *Hp* infection have not been systematically investigated. This study was designed to address this gap by comprehensively examining the infection-specific pattern of L1CAM in vitro in GECs and their corresponding EVs, as well as in vivo in patient-derived gastric biopsies and plasma EVs. By integrating these approaches, we sought to elucidate how *Hp*-induced alterations in L1CAM impact integrin regulation and EMT, thereby contributing to gastric carcinogenesis and aiming to identify a potential *Hp*-associated circulating biomarker signature.

## Materials and methods

### Cell culture and *Hp* strains

AGS (RRID: CVCL_0139) and *Hp* 26695, a cytotoxin-associated gene pathogenicity island (*cag* PAI)-positive strain were obtained from American Type Culture Collection (ATCC; VA, USA), while MKN45 cells and *Hp* 8-1 (a *cag* PAI-negative strain) were acquired under material transfer agreement from the University of Virginia. HFE145, an immortalized non-neoplastic GEC (gifted by Profs. Hassan Ashktorab and Duane T. Smoot) was also used in the study. GECs were routinely validated by short tandem repeat profiling and mycoplasma contamination. Both GECs and bacterial strains were maintained under standard culture conditions [16].

GECs were infected with 10 and 20 multiplicities of infection (MOI) of *Hp* for specified times.

Details of all reagents used in this study are provided in the Supplementary Table 1.

### EV isolation from GECs and characterization

For EV isolation, 20 × 10^6^ AGS cells were grown in 150 mm dishes. Before infection, complete media were replaced with 10% exosome-depleted FBS-supplemented RPMI-1640. Supernatants from uninfected and infected cells were collected after 36 h and sequentially centrifuged at 2000×g, 10 min and 10,000×g, 30 min to remove debris and large particles, respectively. The resulting supernatants were concentrated using a 100 kDa cut-off filter and precipitated using total exosome isolation kit (Invitrogen, Cat. No. 4478359) following manufacturer’s protocol. Pellets were resuspended in PBS and protein content was estimated before downstream experiments.

Following isolation, EVs were characterized by dynamic light scattering (DLS), nanoparticle tracking analysis (NTA) and transmission electron microscopy (TEM) to assess size and morphology. Detailed methods were provided in the supplementary section.

### Immunoblotting

Whole-cell lysates (WCLs) and EVs were prepared in RIPA buffer with protease inhibitors. Equal amounts of protein were loaded onto SDS-PAGE and then transferred to PVDF membranes. Blots were incubated with primary antibodies overnight at 4 °C, followed by HRP-conjugated secondary antibodies at room temperature. Signals were detected using chemiluminescent substrate on a Bio-Rad Chemidoc system. Details of primary and secondary antibodies with dilutions are provided in Supplementary Table 2.

### Human biopsy sample collection and urease test

GC and adjacent healthy tissue samples were obtained from *Hp*-positive patients (30 to 65 years). *Hp* positivity was assessed using the Pylo Dry *Hp* test kit (Halifax Research Laboratory), which is based on urease activity. A color change from yellow to red or pink was considered indicative of an *Hp*-positive result, whereas the absence of a color change was considered indicative of an *Hp*-negative result. Written consents from the patients were obtained following the institutional ethics committee guidelines of NISER. Tissue samples were collected in 4% paraformaldehyde (PFA) and subsequently processed for immunofluorescence microscopy.

### Plasma collection and EV isolation from human volunteers

Peripheral blood samples (4 ml) were collected in EDTA-containing tubes from *Hp*-positive GC patients and sex- and age-matched healthy volunteers (as controls), following institutional ethics guidelines with informed consents. Whole blood was left undisturbed at room temperature for 1 h and only the plasma part was collected. Plasma samples were next centrifuged at 2000×g for 20 min and stored at -80 °C until further processing. For EV isolation, plasma samples were thawed, centrifuged at 10,000×g for 20 min at 4 °C and ultracentrifuged at 110,000×g for 70 min. EV pellets were resuspended in PBS and characterized.

### Hematoxylin-eosin (H&E) staining

Sectioning and H&E staining of human biopsy samples were performed following standard procedure [17]. Brightfield color images were captured using a microscope (Eclipse Ti-U, Nikon).

### L1CAM-stable cell generation and siRNA transfection

L1CAM-stable cells were generated using *L1CAM*-plasmid phL1A-pcDNA3 following a lab-standardized protocol [18] where pcDNA3 served as an empty vector control. Transfections were performed using Lipofectamine 3000 and P3000 reagents for generating stable cell clones and selected using G418.

For L1CAM knockdown, cells were transiently transfected with *NCAM-L1* siRNA and control siRNA using Lipofectamine 3000. Both overexpression and knockdown were validated by immunoblotting. All siRNA-based experiments were performed using a higher bacterial load for a shorter duration (200 MOI for 12 h) as prolonged *Hp* infection following transfection imposed substantial cellular stress. L1CAM level at 20 MOI, 36 h, is comparable to the level at 200 MOI, 12 h.

### GW280264X treatment

AGS cells were co-treated with the ADAM10/17 inhibitor GW280264X (3 µM) to assess proteolytic processing, followed by *Hp* infection (20 MOI, 36 h respectively). Cells were then harvested and analyzed by immunoblotting.

### Colony formation assay

1 × 10^3^ HFE145 cells were plated in 6-well plates pre-treated with EVs (detailed in supplementary materials and methods). Colony formation was observed and fixed with 4% PFA. Colonies were stained using crystal violet and photographed.

### Wound healing assay

Wound healing assay was performed with HFE145 cells pre-treated with EVs. Wounds were made using 200 µl sterile tips. Images were captured at 0, 24, 36 and 48 h time points. Wound healing rate was calculated using the following formula.

$$\text{Wound closure area }\:(\%)\; = \frac{Initial\:wound\:area-Final\:wound\:area}{Initial\:wound\:area}\times\:100$$

### Animal xenograft model

Male NOD/SCID mice (RRID: IMSR_JAX:001303) were used in accordance with NISER animal ethics guidelines. AGS cells (1 × 10^7^, pre-exposed to EVs) were subcutaneously injected into the right flank to induce tumor formation [19]. Once tumors reached 30–60 mm³, mice received 20 µg of EVs (from uninfected or infected conditions) intratumorally every three days for a total of five times [20, 21]. Tumor volumes were measured and calculated as 0.5 × (length × width²). At the end of the experimental period, mice were euthanized and tumors were excised, weighed and photographed. Tumor tissues were fixed in 4% PFA and subjected to immunofluorescence staining to assess L1CAM, Ki-67 and integrins.

### Immunofluorescence/confocal microscopy

Detailed staining protocols for cells and tissues are provided in the supplementary section. Tissue images were acquired using an epifluorescence microscope (Eclipse Ti-U, Nikon) and cell images with a confocal microscope (Sp8, Leica). Images were analyzed using NIS-Element Advanced Research software (Nikon) and Fiji (NIH).

### Statistical significance

Statistical analyses were conducted using GraphPad Prism (version 9; GraphPad Software Inc.). The following methods were applied to assess statistical significance: Student’s t-test, one-way and two-way ANOVA, followed by Tukey’s post hoc analysis wherever applicable. All data were represented as mean ± SEM.

## Results

### *Hp* infection drives coordinated loss of L1CAM in GECs and EVs

**Fig. 1:**
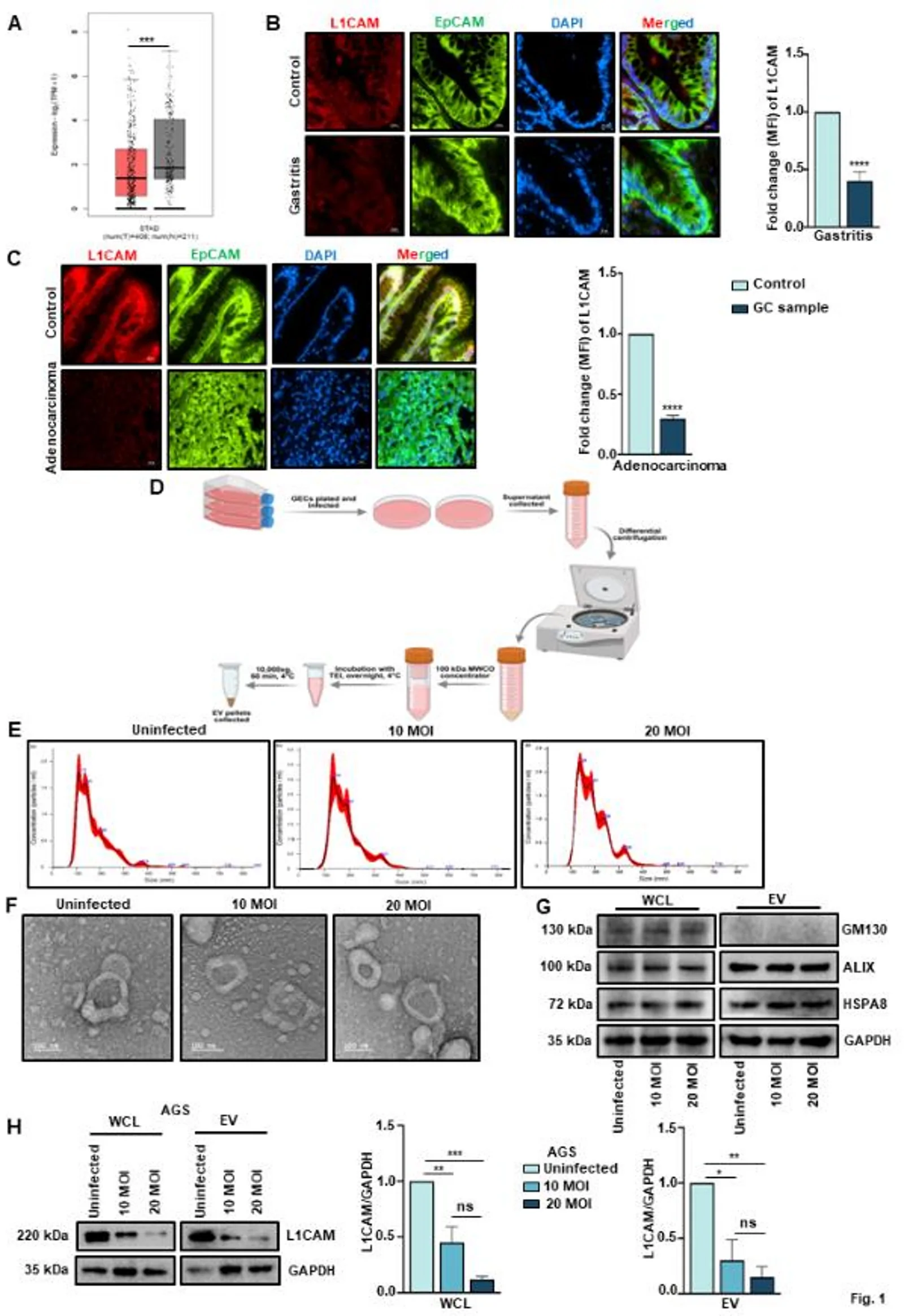
*Hp* infection leads to L1CAM depletion in GECs and EVs. **A** GEPIA2 analysis shows *L1CAM* expression in TCGA-STAD tumor (red) vs. normal (gray) tissues. **B**,** C** Immunofluorescence micrograph showing L1CAM (red) and EpCAM (an epithelial marker, green) in human gastritis (*n* = 5) and adenocarcinoma (*n* = 11) biopsy samples, along with their paired controls. Nuclei were counterstained with DAPI (blue). Bar graphs showing the fold change in mean fluorescence intensity (MFI) of L1CAM in gastritis or adenocarcinoma samples (objective magnification = 60X; scale bar = 20 μm). **D** The schema of EV isolation procedure from GECs combining differential centrifugation, concentration and precipitation-based techniques. **E** NTA analysis and **F** TEM images of EVs isolated from *Hp-*infected/uninfected GECs (scale bar = 100 nm). **G** Western blot analysis of the WCLs and EVs showing the presence of EV-specific markers (GM130 = negative marker, ALIX and HSPA8 = positive markers, GAPDH=loading control). **H** Western blot results displaying L1CAM level in WCLs and EVs obtained from AGS cell lines post *Hp* infection at 10 MOI and 20 MOI. Bar graphs (*n* = 3) indicate fold change of L1CAM relative to GAPDH. GAPDH is the loading control. Statistical significance is determined by one-way ANOVA followed by Tukey’s post hoc analysis and Student’s t-test. Data are represented as mean ± SEM. *, *P* < 0.05; **, *P* < 0.01; ***, *P* < 0.001; ****, *P* < 0.0001; ns, non-significant. **D** was created using BioRender.com

L1CAM plays divergent roles in GC, with evidence of upregulation in some cases and downregulation in others [11, 12]. To gather background information on L1CAM level in GC, we first analyzed its expression in publicly available patient datasets. Box-plot analysis of the TCGA-STAD cohort using GEPIA2 revealed a significant downregulation of *L1CAM* in tumor samples (red) compared with normal gastric samples (gray) [num[T] = 408, num[N] = 211; *p* = 0.001] (Fig. 1A). TIMER2.0 analysis provided further background information on reduced *L1CAM* in tumor samples of STAD dataset, across twelve different tumor subtypes (Supplementary Fig. 1A). Since *Hp* is a known contributor in GC development, it was pertinent to ask whether *Hp* modulated L1CAM level in GC. To address this, immunofluorescence microscopy was performed on *Hp*-positive gastric biopsies obtained from tumor tissues and unaffected peritumoral regions that were *Hp*-negative by rapid urease testing (served as controls). Immunofluorescence micrographs revealed a pronounced reduction in L1CAM in *Hp*-positive gastritis (*n* = 5) and gastric adenocarcinoma tissues (*n* = 11) compared with control tissues (Fig. 1B, C), supporting a potential association between *Hp* infection and L1CAM loss. Given the emerging role of EVs as critical mediators of tumor-associated intercellular communication, we next examined whether L1CAM is altered within EVs derived from *Hp*-infected GECs. Before initiating EV-based studies, AGS, HFE145 and MKN45 cells were evaluated for basal L1CAM level to identify the most suitable model system. Among these, AGS cells exhibited the highest L1CAM expression, whereas HFE145 and MKN45 cells showed comparatively lower levels (Supplementary Fig. 1B). This observation was further supported by Human Protein Atlas data, which also indicated higher L1CAM expression in AGS cells relative to MKN45 cells (Supplementary Fig. 1C). Accordingly, AGS cells were selected for most downstream experiments unless mentioned specifically. For EV isolation, AGS cells were infected with *Hp* at 10 and 20 MOI for 36 h. The schema of EV isolation has been illustrated in Fig. 1D. The isolated EVs were thoroughly characterized as per the MISEV guidelines [22]. The NTA and DLS assays confirmed the size range (100–200 nm) of the isolated EVs (Fig. 1E and Supplementary Fig. 2A), while TEM assessed the morphological features showing the characteristic doughnut-shaped, double-membraned vesicles (Fig. 1F). Additionally, immunoblot analysis confirmed EV purity by detecting canonical EV markers (ALIX and HSPA8) while the absence of the Golgi marker GM130 verified the lack of cellular contamination in EVs (Fig. 1G). Following EV characterization, the status of L1CAM was examined in both WCLs and EVs. *Hp* infection induced a clear, MOI-dependent reduction in full-length (FL) L1CAM in AGS cell-derived lysates and EVs (Fig. 1H). Similarly, a decreasing trend was observed in HFE145 cells and corresponding EVs upon *Hp* infection (Supplementary Fig. 2B). In MKN45 cells, *Hp* infection also led to a reduction of L1CAM in WCLs; however, L1CAM was barely detectable in respective EV samples, concomitant with the intrinsically reduced basal expression of L1CAM in this cell line (Supplementary Fig. 2C). Based on the consistent and robust decrease at 20 MOI, all subsequent experiments were performed under this condition in AGS cells unless stated otherwise. To assess the contribution of the *cag* PAI to *Hp*-induced L1CAM downregulation, AGS cells were infected with *cag* PAI-positive (26695) or *cag* PAI-negative (8-1) *Hp* strains. Immunoblot analysis revealed comparable L1CAM reduction in both WCL and EVs irrespective of *cag* PAI status, indicating *cag*-independent processes (Supplementary Fig. 2D). Collectively, these findings demonstrate that increasing *Hp* infection burden in GECs leads to a progressive and constant loss of L1CAM, both at the cellular level and within EVs.

### *Hp-*infected GEC-EVs promote GC progression both in vitro and in vivo

**Fig. 2:**
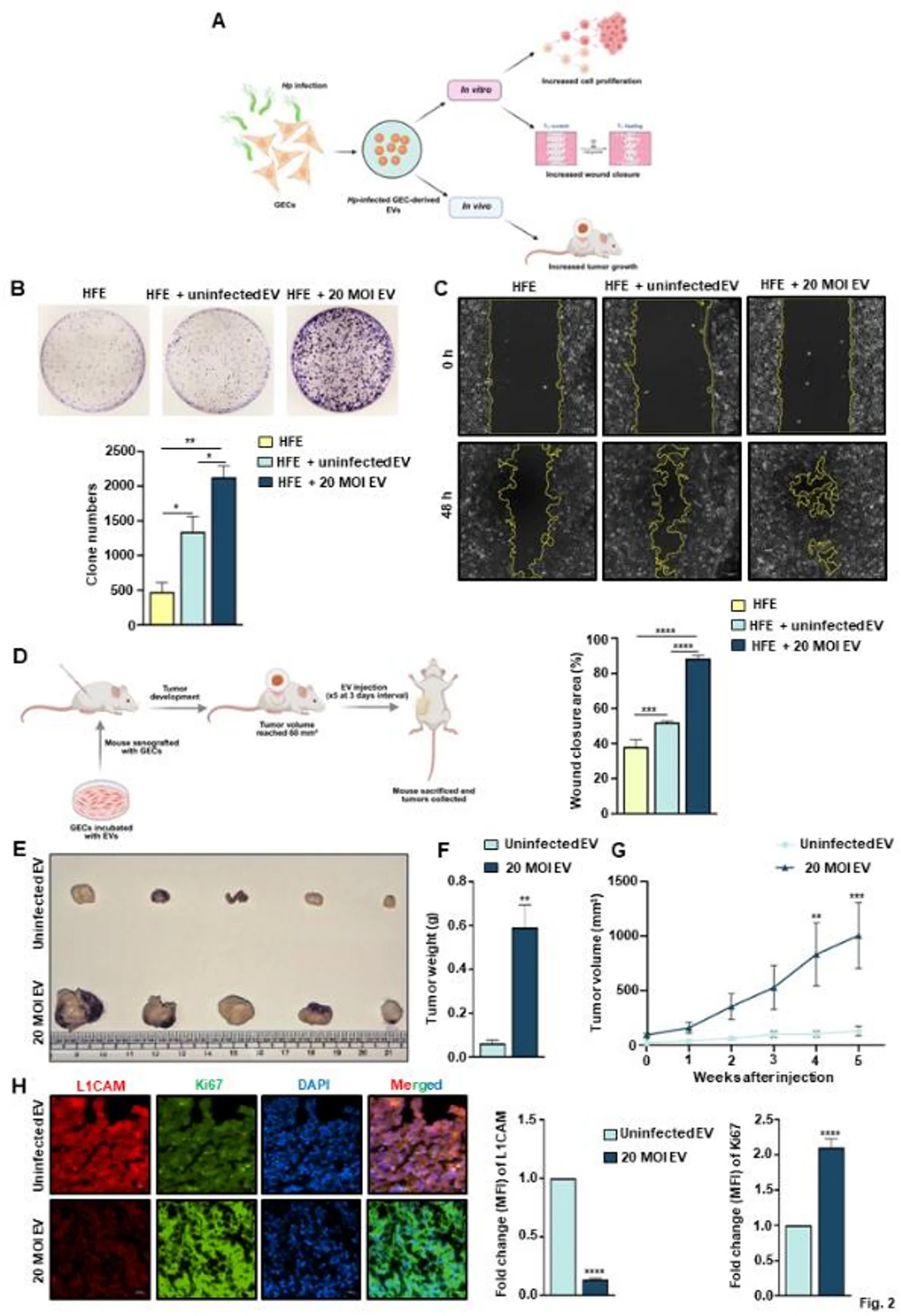
*Hp*-infected GEC-derived EVs cause GC progression both in vitro and in vivo*.* **A** Schematic illustration summarizing the experimental workflow and different assays performed following GEC-EV treatment. **B** Representative image showing colonies in HFE145 cells after incubation with EVs collected from *Hp-*infected or uninfected AGS cells. Bar graph (*n* = 3) depicting the clone numbers in various conditions. **C** Images showing the scratched areas at 0 h and 48 h in HFE145 cells treated with EVs obtained from *Hp-*infected or uninfected AGS cells. Bar graph (*n* = 3) showing the wound closure area (%) (objective magnification = 10X; scale bar = 100 μm). **D** Schema of experimental design showing xenograft strategy and EV-treatment to the xenograft tumors in SCID mice. **E** Image showing tumors excised from mice (*n* = 5). Graphs showing **F** Tumor weight and **G** Tumor volume comparison between infected and uninfected groups. **H** Immunofluorescence micrographs (*n* = 5) of tumor tissues excised from SCID mice showing expression of L1CAM (red) and Ki67 (green) in infected and uninfected conditions. DAPI stains the nuclei (blue). Ki67 is used as a proliferation marker. Bar graphs showing the fold change in MFI of L1CAM and Ki67 in tissue samples (objective magnification = 60X; scale bar = 20 μm). Statistical significance is determined by one-way ANOVA and two-way ANOVA for tumor volume measurements followed by Tukey’s post hoc analysis and Student’s t-test. Data are represented as mean ± SEM. *, *P* < 0.05; **, *P* < 0.01; ***, *P* < 0.001; ****, *P* < 0.0001. **A**,** D** were created using BioRender.com

By transferring bioactive components, including proteins, EVs facilitate crosstalk between malignant cells and adjacent non-malignant cells [23]. This contributes to the dynamic reprogramming of the TME, promoting tumor growth and metastasis. The experimental workflow and the assays performed following GEC-EV treatment are summarized schematically in Fig. 2A. To assess whether EVs from *Hp*-infected AGS influence non-cancerous GECs, HFE145 cells were treated with EVs from uninfected and infected AGS cells. Infected AGS-EVs significantly enhanced colony formation (Fig. 2B) and wound healing ability (Fig. 2C) of HFE145 cells compared to uninfected or untreated controls, indicating EV-mediated pro-proliferative effects. EMT markers were then analyzed to further confirm the molecular players associated with the phenotypic changes. *Hp*-infected AGS cells led to the upregulation of N-cadherin, Snail and Twist1 (Supplementary Fig. 3A), while E-cadherin was absent due to its null-expression in AGS cells [24]. Importantly, *Hp*-driven EMT induction was further enhanced by silencing of *L1CAM* (Supplementary Fig. 3B). These findings indicated towards an EMT-suppressing role of FL-L1CAM.

The tumor-promoting effects of *Hp*-infected GEC-EVs were further evaluated in vivo using a subcutaneous xenograft model in NOD/SCID mice (Fig. 2D). Tumors arising from the cells treated with EVs derived from *Hp*-infected AGS cells exhibited significantly greater volume and weight compared to those treated with EVs from uninfected cells (Fig. 2E-G). Immunofluorescence microscopy of xenograft tissues from infected EV-treated mice showed significantly reduced L1CAM and increased Ki67 compared to the uninfected group (Fig. 2H). Together, these findings demonstrated that EVs from *Hp*-infected GECs, enhanced cell proliferation and migration in vitro and drove tumor growth in vivo.

### *L1CAM*-overexpressing cell-derived EVs suppress *Hp*-mediated GC progression both in vitro and in vivo

**Fig. 3:**
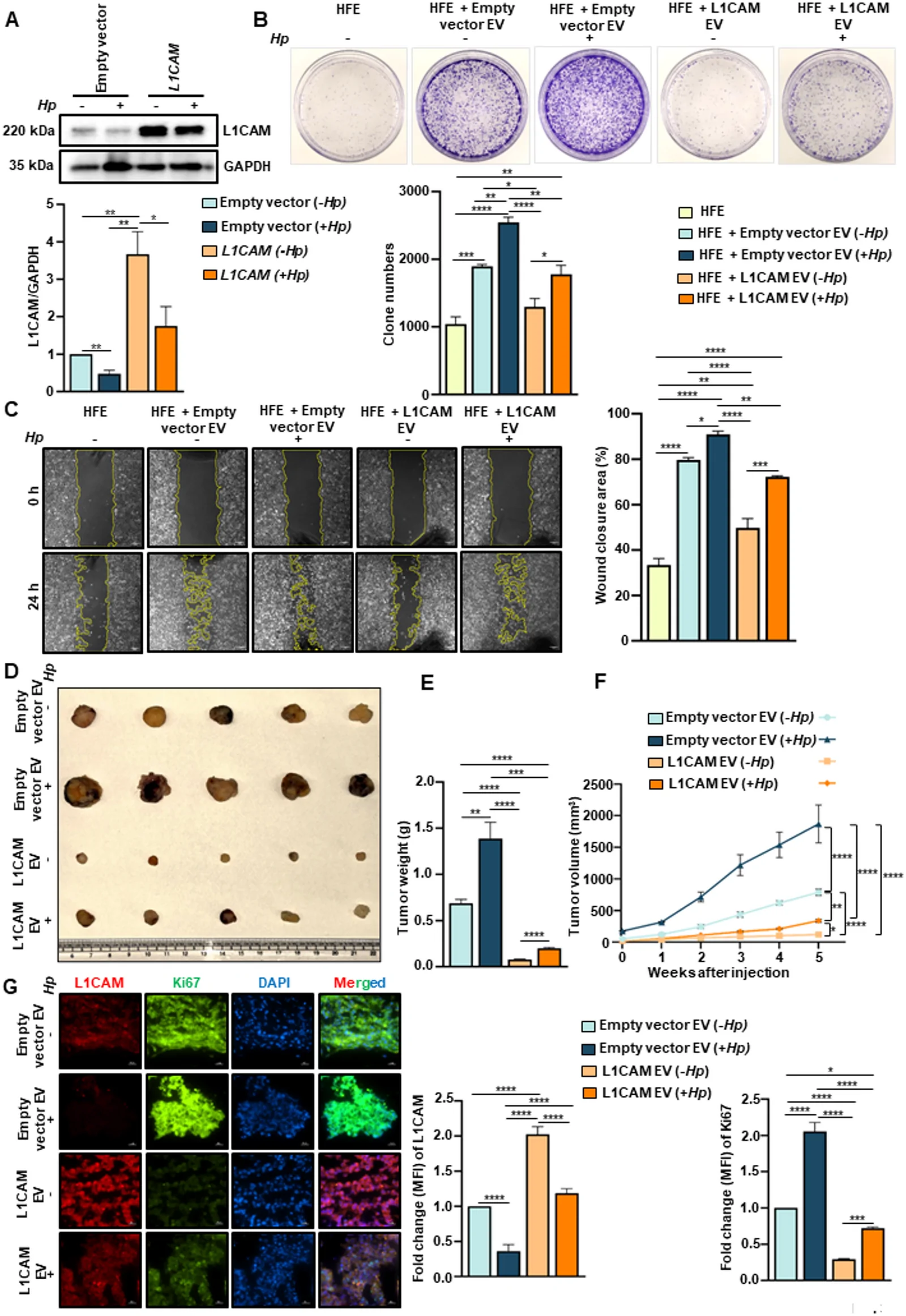
*Hp*-driven L1CAM downregulation in GEC-EVs promotes GC progression which is suppressed by L1CAM-EVs. **A** AGS cells transfected with phL1A-pcDNA3 plasmid or empty vector plasmid showing status of FL-L1CAM with/without infection after immunoblotting. GAPDH is the loading control. Bar graph (*n* = 3) shows the fold change in L1CAM relative to GAPDH. **B** Images representing colonies in HFE145 cells upon *Hp*-infected or -uninfected L1CAM- and empty vector- stable cell-derived EV treatment. Accompanying graph (*n* = 3) represents the clone numbers. **C** Bright field micrographs showing scratched areas at 0 h and 48 h in HFE145 cells treated with EVs derived from infected or uninfected L1CAM- and empty vector-stable cells. Bar graph (*n* = 3) shows the percent change in wound closure area (objective magnification = 10X; scale bar = 100 μm). **D **Photograph of tumors (*n* = 5) excised from SCID mice after xenograft with AGS cells, treated with EVs derived from L1CAM/empty vector-expressing cells with/without infection. **E** Graph representing tumor weight of each group. **F** Continuous plot showing tumor volume growth curve of xenograft tumors in SCID mice, measured for 5 weeks. **G** Immunofluorescence micrographs of tumor tissues from SCID mice (*n* = 5) showing L1CAM (red) and Ki67 (green). DAPI (blue) stains nuclei. Bar graphs showing the fold change in MFI of L1CAM and Ki67 in tissue samples (objective magnification = 10X; scale bar = 100 μm). Statistical significance is determined by one-way ANOVA and two-way ANOVA for tumor volume measurements followed by Tukey’s post hoc analysis. Data are represented as mean ± SEM. *, *P* < 0.05; **, *P* < 0.01; ***, *P* < 0.001; ****, *P* < 0.0001; ns, non-significant.

Given the *Hp*-induced loss of L1CAM and the pro-tumorigenic effects of EVs derived from infected cells observed in our study, we next investigated whether the restoration of L1CAM expression could modulate *Hp*- and EV-mediated GC progression. For this reason, AGS cells were stably transfected with *L1CAM* and its corresponding empty vector. Despite overexpression, *Hp* infection decreased the FL-L1CAM in *L1CAM*-overexpressed cells as verified by immunoblotting (Fig. 3A). Next, the functional impact of EVs derived from these groups were examined. EVs from *Hp*-infected cells, particularly those from the empty vector-transfected cells with the lowest L1CAM expression, induced maximal colony formation and migration of recipient HFE145 cells (Fig. 3B, C). In contrast, EVs from uninfected cells (both from *L1CAM*-overexpressing and empty vector cells), which retained higher L1CAM, significantly suppressed these pro-tumorigenic behaviours. Notably, EVs from *L1CAM*-overexpressing uninfected cells exerted the strongest inhibitory effects, highlighting the functional importance of L1CAM abundance within EVs. These in vitro observations were next corroborated in vivo using a NOD/SCID xenograft model to determine whether *Hp*-driven alterations in EV-associated L1CAM similarly influenced tumor growth. Consistent with in vitro findings, EVs derived from *Hp*-infected cells markedly promoted tumor growth, as reflected by increased tumor volume and weight, irrespective of enforced L1CAM expression. This effect was most pronounced in mice receiving EVs from *Hp*-infected empty vector cells, which contained minimal L1CAM. Conversely, EVs from uninfected *L1CAM*-overexpressing cells significantly limited tumor development, demonstrating that preservation of L1CAM in the absence of *Hp* infection attenuated EV-mediated tumor progression (Fig. 3D-F). Congruent with these growth patterns, tumors treated with EVs from *Hp*-infected cells exhibited elevated Ki67 expression accompanied by reduced L1CAM level, whereas tumors receiving EVs from uninfected *L1CAM*-overexpressing cells showed diminished proliferative indices with relative maintenance of L1CAM expression (Fig. 3G). Collectively, these results demonstrate that *Hp* infection induces a robust reduction in FL-L1CAM expression, even under overexpression conditions. L1CAM downregulation thus emerges as a critical downstream consequence of *Hp* infection that functionally contributes to gastric tumor growth and aggressiveness.

### *Hp* infection-associated loss of FL-L1CAM is mirrored in circulating EVs of GC patients 

**Fig. 4:**
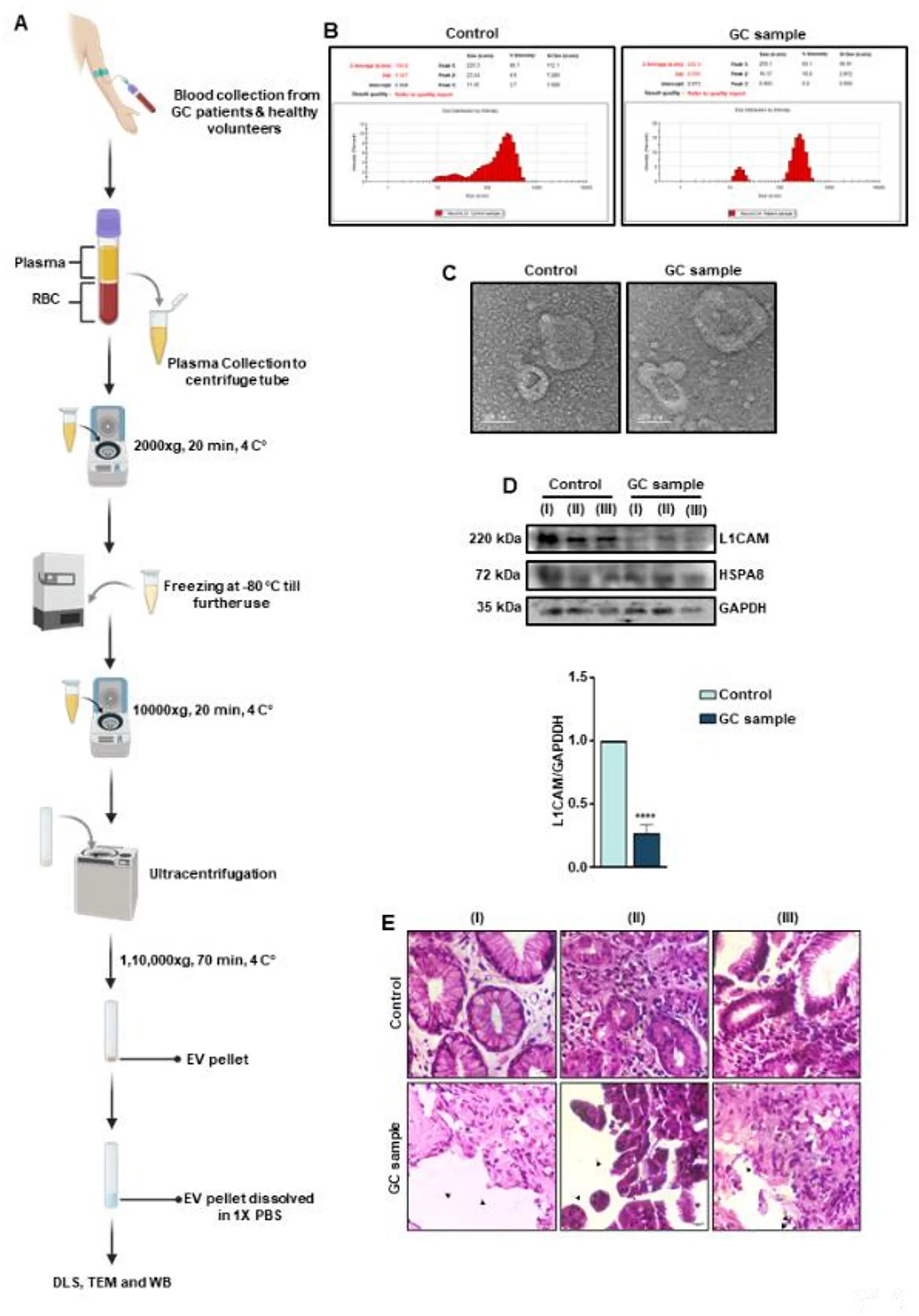
FL-L1CAM is downregulated in plasma-EV samples obtained from *Hp*-positive GC patients. **A** The schematic shows the procedure for EV isolation from plasma of GC patients/healthy volunteers. **B** DLS analysis and **C** TEM images of plasma-derived EVs (scale bar = 100 nm). **D** Immunoblots show FL-L1CAM in plasma-derived EVs. HSPA8 serves as the positive marker for EV. GAPDH serves as a loading control. Bar graph (*n* = 12) displays the fold change in L1CAM. **E** Representative H&E images of tissues from *Hp*-positive GC patients along with adjacent uninvolved tissues showing presence of *Hp* (indicated with black pointers; objective magnification = 30X; Scale bar = 50 μm). Statistical significance is determined by Student’s t-test. Data are represented as mean ± SEM. ****, *P* < 0.0001. **A** created using BioRender.com

Given the persistent reduction of FL-L1CAM observed upon *Hp* infection, we next examined whether this alteration could be detected in plasma-derived EVs from *Hp*-positive GC patients. For this purpose, EVs were isolated from plasma samples obtained from *Hp*-positive GC patients and sex- and age-matched healthy volunteers using ultracentrifugation, schematically illustrated in Fig. 4A. DLS and TEM analyses confirmed that the isolated vesicles were within the expected size range (150–250 nm) and displayed characteristic EV morphology (Fig. 4B, C). Immunoblotting revealed significantly reduced FL-L1CAM protein in plasma-EVs from *Hp*-positive GC patients compared with plasma-EVs from *Hp*-negative healthy volunteers (Fig. 4D). H&E staining of corresponding gastric biopsies showed the presence of *Hp* (Fig. 4E). Findings from both in vitro studies and clinical specimens indicated that the loss of L1CAM may represent a defining molecular feature of *Hp*-associated GC. Moreover, the downregulation of L1CAM reflected in the plasma-derived EVs, presented L1CAM as a minimally invasive biomarker for the detection of *Hp*-positive GC patients.

### FL-L1CAM loss is associated with decreased integrin αVβ1 in *Hp*-infected GECs

**Fig. 5:**
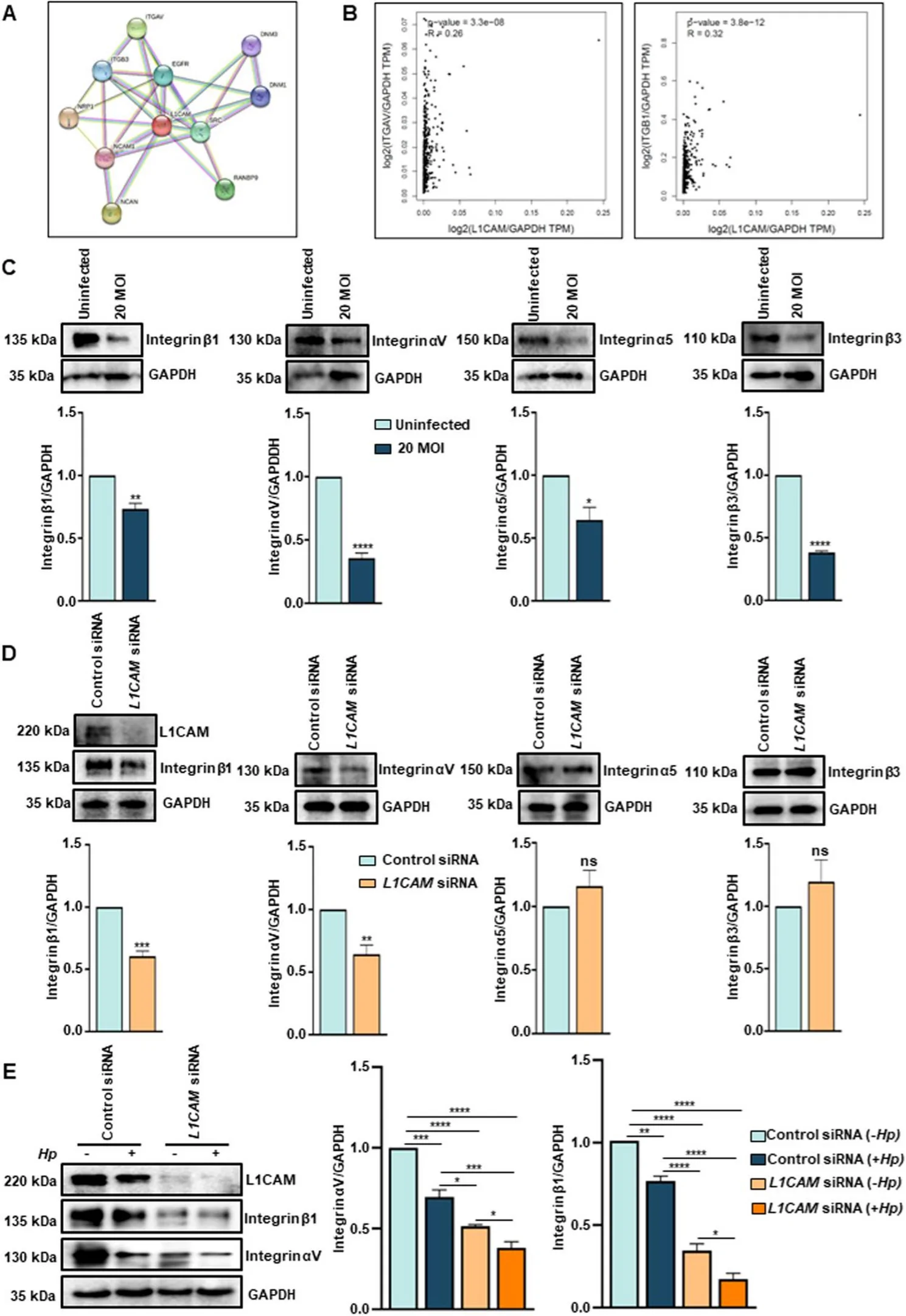
L1CAM suppression downregulates αV and β1 integrins in *Hp*-infected GECs. **A** STRING analysis reveals the L1CAM-interacting integrins. **B** Plots showing correlation analysis between *L1CAM* and *ITGAV* or *ITGB1* using the TCGA-STAD database in GEPIA2. **C** Immunoblots show integrin β1, αV, α5 and β3 in AGS with/without infection. Graphs (*n* = 3) show the fold change in integrins relative to GAPDH. **D** Representative blots indicate β1, αV, α5 and β3 integrins in *L1CAM*-suppressed AGS cells. Graphs (*n* = 3) show the fold change in integrins relative to GAPDH. **E** L1CAM, αV and β1 integrin levels in infected/uninfected *L1CAM* siRNA and control siRNA-transfected AGS cells. Bar graphs (*n* = 3) present fold change in integrin αV and integrin β1 levels relative to GAPDH. GAPDH is the loading control. Statistical significance is determined by the Student’s t-test and one-way ANOVA followed by Tukey’s post hoc analysis. Data are represented as mean ± SEM. *, *P* < 0.05; **, *P* < 0.01; ***, *P* < 0.001; ****, *P* < 0.0001. ns, non-significant

**Fig. 6:**
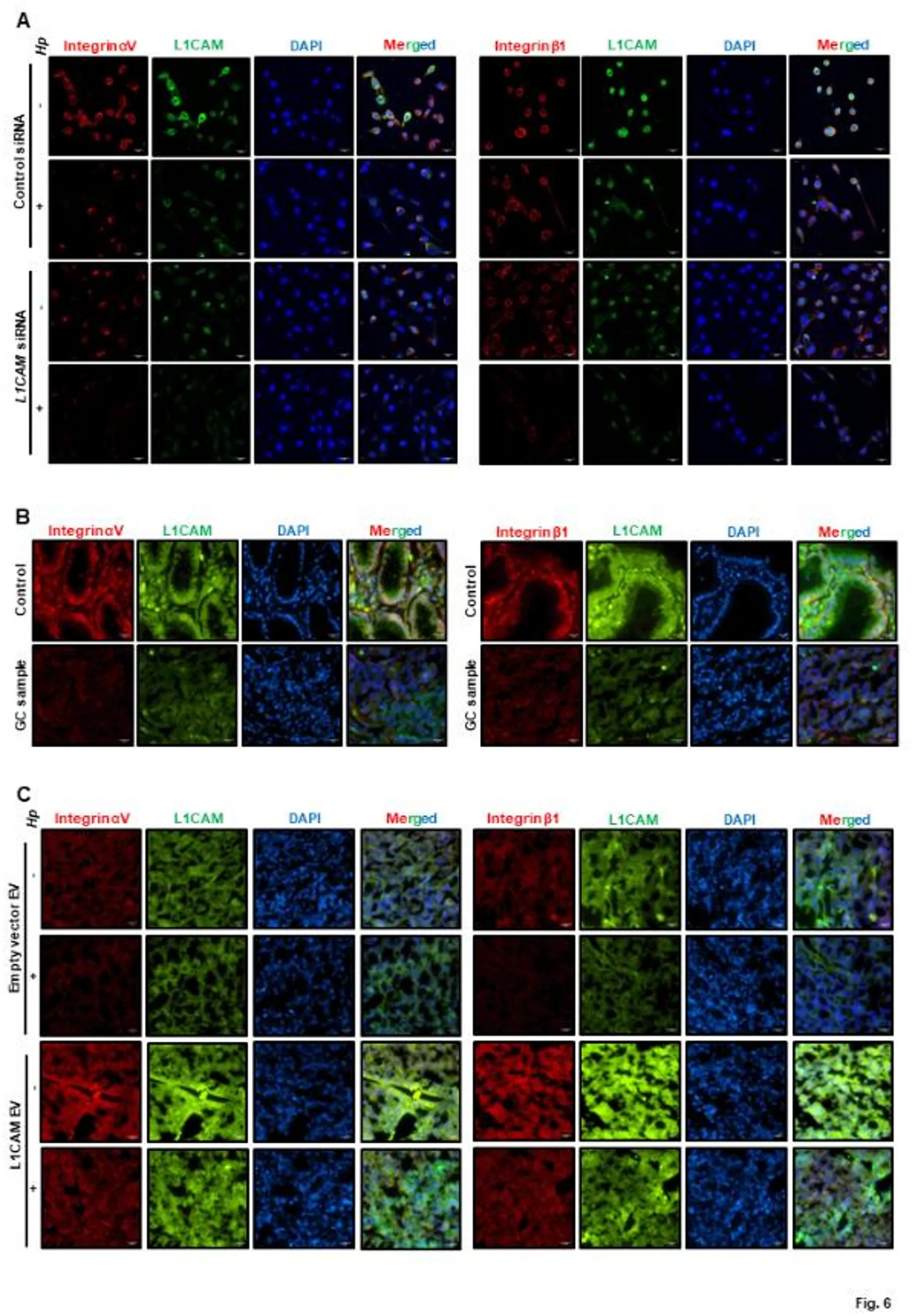
Downregulation of αVβ1 integrins in *Hp*-positive cells and tissues and in infected GEC-EV-treated mouse xenografts. **A** Confocal images (*n *= 3) show L1CAM (green) and αV/β1 integrins (red) in infected/uninfected *L1CAM* siRNA and control siRNA-transfected AGS cells. DAPI (blue) stains nuclei. **B **Immunofluorescence micrographs (*n* = 3) show L1CAM (green) and αV/β1 integrins (red) in *Hp*-positive GC biopsies along with *Hp*-negative peritumoral control tissues obtained from patients. Nuclei stained with DAPI (blue). **C** Fluorescence micrographs showing L1CAM (green) and αV/β1 integrin (red) levels in tumor tissues obtained after xenograft in SCID mice (*n* = 5). For **A**, objective magnification = 63X; scale bar = 20 μm and for **B** and **C**, objective magnification = 60X; scale bar = 20 μm

Integrins are key regulators of cell adhesion, migration and tumor invasiveness and are well-recognized contributors to *Hp*-mediated gastric carcinogenesis [25–27]. Disruption of integrin-dependent adhesion is a hallmark of EMT and GC progression [25]. L1CAM, a cell adhesion molecule essential for maintaining epithelial integrity, has been proposed to functionally interact with integrin signaling networks [28, 29]; however, this crosstalk remains underexplored in the context of *Hp* infection. Supported by prior reports [30], interaction analyses (Fig. 5A) and clinical correlation datasets, integrin αV, β1, α5 and β3 were therefore selected to investigate whether *Hp*-induced loss of L1CAM is accompanied by coordinated alterations in integrin expression. Correlation analysis using GEPIA2 revealed positive associations of L1CAM with ITGAV and ITGB1 (*R* = 0.26 and 0.32, respectively) (Fig. 5B) in contrast to comparatively weaker associations with ITGA5 and ITGB3 (*R* = 0.13 and 0.21) (Supplementary Fig. 4A). In vitro experiments were performed to validate the observed correlation between integrins and L1CAM. Immunoblotting revealed a significant decrease in all four integrins in *Hp*-infected AGS cells. (Fig. 5C). Interestingly, siRNA-mediated L1CAM depletion selectively decreased αV and β1 integrins with no effect on α5 and β3 (Fig. 5D). To further evaluate the *Hp*-dependent modulation of αV and β1 integrins in the absence of L1CAM, L1CAM-silenced cells were challenged with *Hp*. Immunoblotting showed a significant downregulation of αV and β1 integrins in *Hp*-infected *L1CAM* siRNA-transfected cells compared with both uninfected L1CAM-silenced cells and control siRNA cells (Fig. 5E), corroborating the confocal microscopic analyses (Fig. 6A and Supplementary Fig. 4B). Notably, the combined effect of *Hp* infection and L1CAM knockdown led to a pronounced reduction of αV and β1 integrins compared to that observed in either condition alone. *Hp*-positive GC patient biopsies also revealed concurrent downregulation of L1CAM, integrin αV and integrin β1 (Fig. 6B and Supplementary Fig. 4C). Conversely, xenografts treated with EVs from *L1CAM*-overexpressing cells displayed elevated αV and β1 relative to empty vector groups under uninfected conditions, whereas EVs from infected cells caused a pronounced decrease in both the integrins (Fig. 6C and Supplementary Fig. 4D). Therefore, these findings suggest that FL-L1CAM is associated with the maintenance of αV and β1 integrins and that its loss during *Hp* infection might contribute to the observed reduction of these integrins in GECs.

### *Hp* infection-mediated L1CAM loss is attributed to proteolytic cleavage

**Fig. 7:**
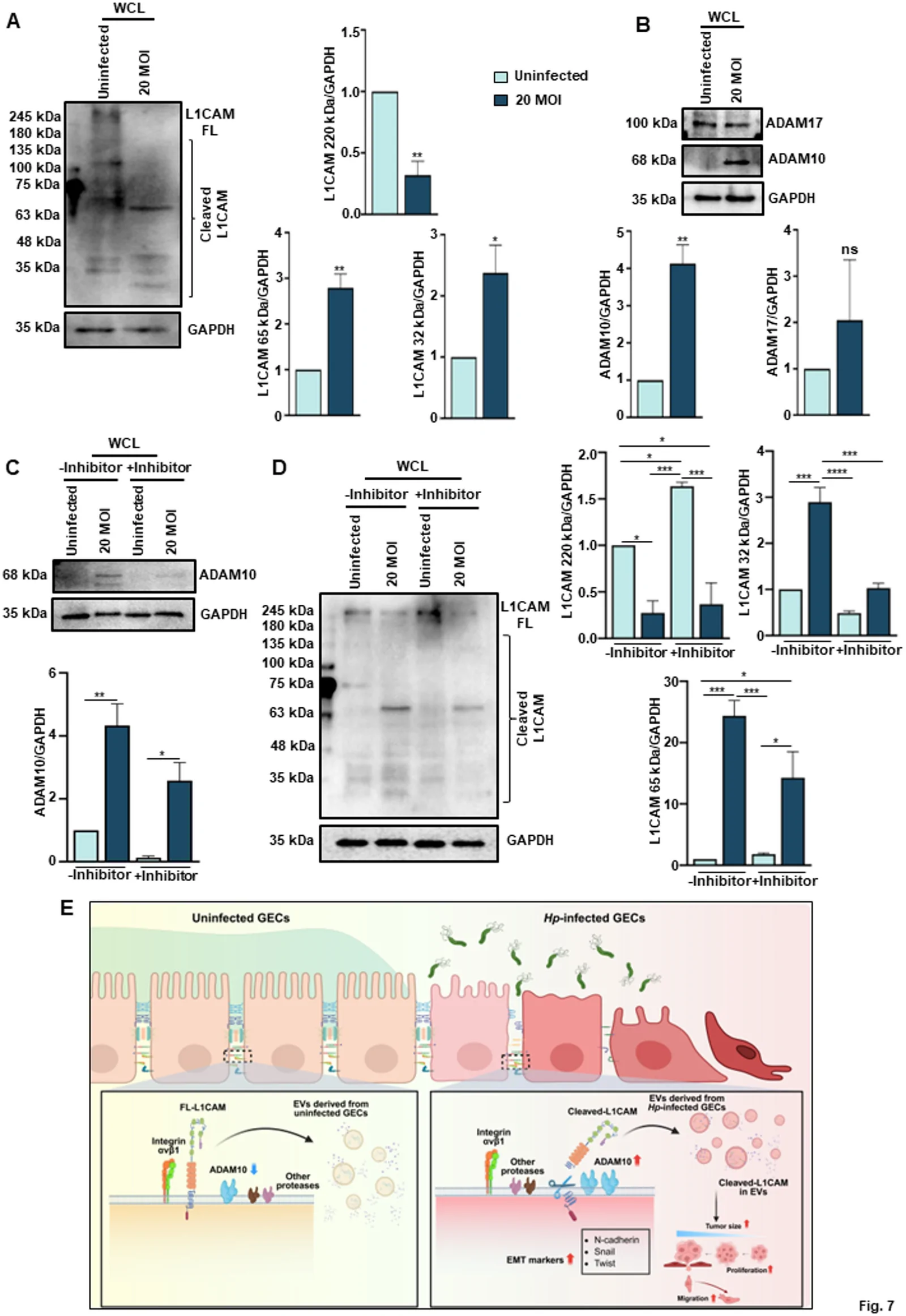
*Hp* infection triggers ADAM10-dependent L1CAM proteolysis. **A** Detection of FL and proteolyzed L1CAM bands in AGS cells under uninfected/infected conditions. Bar graphs showing the fold change of ~ 220 kDa, ~ 65 kDa and ~ 32 kDa L1CAM fragments relative to GAPDH (*n* =3). **B** Immunoblot showing ADAM10 and ADAM17 status in AGS WCLs post *Hp* infection. Graphs (*n *= 3) display fold change in ADAM10 and ADAM17 relative to GAPDH. **C** Western blot showing ADAM10 in AGS WCLs post *Hp* infection along with ADAM10/17 inhibitor (GW280264X) treatment. Bar graphs (*n* = 3) show ADAM10 fold change relative to GAPDH in both inhibitor treated and untreated groups. **D** Immunoblot analysis of FL and proteolytically processed L1CAM in AGS cells with or without *Hp* infection, in the presence or absence of ADAM10/17 inhibitor. Accompanying graph (*n* = 3) represents ~ 220 kDa, ~ 65 kDa and ~ 32 kDa L1CAM fold change relative to GAPDH. GAPDH is the loading control. **E** Schematic illustration of the L1CAM-ADAM10-αVβ1 integrin axis in *Hp*-mediated gastric carcinogenesis. *Hp* infection of GECs induces ADAM10-dependent proteolytic cleavage of FL-L1CAM, resulting in reduced αVβ1 integrin expression and promotion of EMT. *Hp*-induced EMT is depicted by altered expression of EMT markers. In parallel, EVs enriched in L1CAM suppress proliferation and migration of non-cancerous GECs in vitro and limit tumor growth in vivo, whereas EVs derived from *Hp*-infected GECs reverse this effect. Statistical significance is determined by the Student’s t-test and one-way ANOVA followed by Tukey’s post hoc analysis. Data are represented as mean ± SEM. *, *P *< 0.05; **, *P* < 0.01; ***, *P* < 0.001; ****, *P* < 0.0001; ns, non-significant; FL, full-length. **E** was created using BioRender.com

Transmembrane proteins are highly susceptible to proteolytic shedding, a process that cleaves and releases their extracellular domain into a soluble form, thereby regulating protein stability and functional activity [31]. Therefore, we investigated whether *Hp* infection promotes proteolytic processing of L1CAM. To address this, full-length immunoblots were analyzed to assess L1CAM processing. Immunoblotting of WCLs revealed the appearance of prominent cleavage fragments at approximately 65 and 32 kDa in *Hp*-infected samples, whereas the 220 kDa FL-L1CAM band was predominantly detected in uninfected lanes (Fig. 7A). *Hp* infection activates multiple proteases including ADAM family members and prior studies revealed L1CAM as a known substrate for ADAM proteases (especially ADAM10 and 17) [32–35]. Our results showed the significant upregulation of ADAM10 in *Hp*-infected cells, whereas ADAM17 increase was not statistically significant in WCLs (Fig. 7B). To determine whether ADAM10 activity contributes to L1CAM cleavage, cells were treated with an ADAM10/17 inhibitor. Treatment with the inhibitor markedly downregulated ADAM10 in infected cells (Fig. 7C) and partially attenuated L1CAM cleavage, as evident from decreased 65 and 32 kDa fragments in WCLs and 32 kDa fragments in secreted EVs (Fig. 7D and Supplementary Fig. 5A). ADAM inhibition preserved FL-L1CAM only under the uninfected condition. Notably, inhibition of ADAM was insufficient to restore the FL-L1CAM in the presence of ongoing *Hp* infection. Together, these findings demonstrate that *Hp* infection induces ADAM10 upregulation and promotes proteolytic cleavage of FL-L1CAM, identifying ADAM10-mediated L1CAM processing as one of the characteristic feature of *Hp*-driven gastric carcinogenic signaling. A brief overview of the entire study is provided in Fig. 7E.

## Discussion

*Hp* infection initiates a cascade of oncogenic events that disrupt epithelial homeostasis and drive GC progression [1]. Despite *Hp* being the primary etiological agent causing GC, the status and regulation of L1CAM in the context of *Hp *infection-mediated GC have remained completely unexplored. This study reports for the first time that *Hp* infection in GECs induces a marked downregulation of the FL-L1CAM. EVs derived from L1CAM-stable cells exert potent tumor-suppressive effects both in vitro and in vivo. These suppressive effects are partly reversed upon *Hp* infection, indicating towards a mechanism by which *Hp* overrides L1CAM-mediated tumor restraining ability. Further findings confirm that *Hp* contributes to EMT not only through canonical mechanism but also by altering key adhesion molecules involved in epithelial integrity. The loss of FL-L1CAM closely paralleled the decrement of αV and β1 integrins which are known to influence cell adhesion. Together, these findings demonstrate the dynamic regulation of *Hp*-mediated GC by L1CAM downregulation. This study identifies *Hp*-mediated marked downregulation of FL-L1CAM in GECs and their corresponding EVs. *Hp-*positive GC patient biopsies also show FL-L1CAM downregulation. Furthermore, comparison of *cag* PAI-positive and *cag* PAI-negative *Hp* strains suggests that virulence factors beyond CagA [36] may contribute to L1CAM loss and disease progression.

In the context of *Hp* infection, loss of L1CAM may constitute a critical breach in epithelial integrity that predisposes to cancer invasiveness. As a transmembrane adhesion molecule L1CAM is central in maintaining epithelial cohesion by interacting with integrin molecules [35]. Thus, its downregulation is likely to destabilize adhesion complexes and facilitate mesenchymal reprogramming. Consistent with reports that *Hp* perturbs integrin stability during disease progression [27], the reduction of FL-L1CAM closely parallels the decline of integrins, indicating coordinated disruption of the L1CAM-integrin adhesion axis. Therefore, L1CAM depletion in the current scenario of *Hp* infection can be interpreted not merely as a molecular alteration but as a functional disruption of epithelial integrity, marked by upregulation of canonical EMT drivers such as Snail, Twist1 and N-cadherin, ultimately contributing to infection-associated invasiveness of GC.

L1CAM, through its ectodomains, mediate homophilic as well as heterophilic interactions [14]. Importantly, these interactions can be disrupted by proteolytic cleavages within the ectodomain of L1CAM, generating soluble and membrane-associated fragments with distinct biological activities [37, 38]. Previous studies indicate that L1CAM ectodomain shedding is differentially regulated by ADAM proteases [31]. Constitutive L1CAM cleavage is primarily mediated by ADAM10, whereas phorbol myristate acetate stimulation shifts processing toward ADAM17 [35]. In addition, Gutwein et al. report ADAM10-dependent L1CAM cleavage at the cell surface and in released membrane vesicles, underscoring the context-dependent control of its shedding [37]. Previous studies have also shown elevated epithelial expression of ADAM10 and ADAM17 in *Hp*-infected gastric biopsies and ADAM-driven ectodomain shedding of various proteins as a potential contributor to disease pathogenesis [32]. Accordingly, the status of both ADAM10 and ADAM17 have been analyzed, followed by assessment with the dual ADAM10/17 inhibitor GW280264X. Interestingly, under *Hp* infection, only ADAM10 gets markedly upregulated, whereas ADAM17 upregulation is not significant, indicating a predominant role for ADAM10 in this context. ADAM10-mediated cleavage of L1CAM generates a ~ 32 kDa fragment, consistent with prior reports [38]. In addition, multiple bands are observed, including a predominant band at approximately 65 kDa, suggesting the potential involvement of additional proteases in L1CAM processing. Interestingly, treatment with the inhibitor GW280264X does not fully restore L1CAM level. As reported by Maretzky et al., GW280264X functions as a broad-spectrum inhibitor with preferential activity against ADAM17 and comparatively weaker inhibition of ADAM10 [35]. In addition, several infection‑induced host signaling events are known to initiate rapidly, within 5 min to 1 h post‑infection [39], whereas optimal inhibitory activity of GW280264X typically requires prolonged exposure, up to 48 h [40]. Since we collected EVs at 36 h, we could not extend inhibitor treatment beyond this duration, which may not have been sufficient for complete suppression of ADAM10 activity.

L1CAM, via its FNIII and Ig domains, heterophilically interacts with integrins [28, 29, 41, 42]. Although our study demonstrates a significant reduction of integrin αV, α5, β3 and β1 upon *Hp* infection, siRNA-mediated downregulation of L1CAM confirms that only integrins αV and β1 are associated with L1CAM loss, identifying these as potential downstream effectors. Proteolytic cleavage of the L1CAM ectodomain by ADAM or other proteases has been reported to disrupt these interactions [29, 35]. This concurrent decline may reflect ADAM10-mediated proteolytic processing of L1CAM during infection, leading to destabilization of integrin-associated adhesion complexes. Whether the observed reduction in αV and β1 integrins results from direct regulation by L1CAM or secondary cellular remodeling, that warrants further investigation. It would be interesting to dive deeper into the integrin-mediated regulation of the proteases acting on L1CAM. Conversely, understanding the effect of L1CAM fragments on the activity of integrins may provide novel insights into *Hp*-mediated GC progression.

Previous literature reported that exosomes serve as a novel platform for the cellular export of proteolyzed L1CAM [31]. In line with these observations, our findings indicate that *Hp* infection promotes ADAM10-mediated L1CAM proteolysis and its subsequent release in EVs [37]. The shed ectodomain of L1CAM has been shown to exhibit distinct pro-migratory functions [38]. In this context, our findings show that EVs derived from infected cells induced migration of non-cancerous GECs, suggesting a paracrine mechanism of epithelial modulation.

Though a prior article contended about the specificity of L1CAM antibody [43], the use of the clone 2C2 antibody targeting the C-terminal epitope in our experiments ensures the detection of FL- as well as the fragmented L1CAM. The 32 kDa transmembrane fragment, together with a ~ 200 kDa fragment, both displaying differential expression patterns, gets predominantly enriched in EV samples. The presence of these distinct L1CAM fragments within EVs demands further investigation to reveal their functional significance following vesicular transfer. Also, the loss of FL-L1CAM in plasma-derived EVs offers a compelling glimpse into the potential of L1CAM as a minimally-invasive circulating biomarker for *Hp*-mediated GC. Defining the biological roles of EV-associated L1CAM fragments may open new avenues of research, as proteolytic cleavage products often have altered specificity in signaling events than their FL counterparts and may differentially influence disease progression.

## Conclusion

In conclusion, this study identifies loss of FL-L1CAM as a defining molecular event in *Hp*-associated GC. Notably, *Hp* infection of GECs and their respective EVs demonstrates marked loss of FL-L1CAM, linking epithelial adhesion disruption to αVβ1 integrin downregulation, EMT induction and EV-driven pro-tumorigenic signaling. The consistent reduction of FL-L1CAM in tumor tissues and plasma-derived EVs from *Hp*-positive GC patients underscores its potential as a minimally invasive biomarker. Our findings show that ADAM10 contributes to the proteolytic processing and loss of FL-L1CAM during *Hp* infection, although the involvement of additional proteases cannot be excluded. A detailed mechanistic interplay within the L1CAM-ADAM10-αVβ1 integrin axis remains to be fully elucidated. Future studies focusing on fragment-specific signaling functions, longitudinal patient validation and therapeutic targeting of this pathway will be essential to translate these findings into clinical applications.

## Supplementary Information

Supplementary Material 1.

Supplementary Material 2.

## Data Availability

The data generated in this study are available upon request from the corresponding author.

## Abbreviations

ADAM10/17: A disintegrin and metalloproteinase 10/17
*cag* PAI: Cytotoxin-associated gene pathogenicity island
DLS: Dynamic light scattering
EMT: Epithelial-mesenchymal transition
EV: Extracellular vesicle
FL: Full-length
FNIII: Fibronectin-typeIII
GC: Gastric cancer
GEC: Gastric epithelial cells
*Hp*: *Helicobacter pylori*
Ig: Immunoglobulin
L1CAM: L1 cell adhesion molecule
MOI: Multiplicity of infection
NTA: Nanoparticle tracking analysis
TEM: Transmission electron microscopy
TME: Tumor microenvironment
WCL: Whole cell lysate
