# Supplementary material for: Loss of L1CAM from gastric epithelial cells and extracellular vesicles promotes Helicobacter pylori-driven cancer progression and invasiveness
Source: Cell Commun Signal. 2026 Jul 25;24:484. doi: 10.1186/s12964-026-03087-5 (PMC13560308; doi:10.1186/s12964-026-03087-5)
Supplement: Supplementary file 1 [file 12964_2026_3087_MOESM1_ESM.docx]

**Supplementary materials and methods**

**Dynamic light scattering (DLS)**

DLS was performed to study the size of EVs using the Zetasizer Nano ZS system (Malvern Instruments). 10 µl EV sample was diluted in 990 µl PBS. DTS 2.0 software was used for data acquisition and analysis.

**Nanoparticle tracking analysis (NTA)**

EV concentration and size distribution were assessed using NTA. The EV samples were analyzed using the NanoSight NS300 (Malvern Instruments) system. Data acquisition and analysis were carried out using NTA v3.00 software, following the manufacturer's instruction.

**Transmission electron microscopy (TEM)**

EV samples were adsorbed onto carbon films on 300 mesh copper grids for 20 min at room temperature (RT). Excess liquid was removed by gently soaking with blotting paper. The grids were then stained with 2.5% uranyl acetate for 10 min, followed by a brief wash. Grids were again stained for 2 min and dried. The grids were imaged using a JEOL-JEM 1230 microscope to visualize EV morphology and size.

**Bioinformatics analysis**

The Gene Expression Profiling Interactive Analysis 2 (GEPIA2) ([http://gepia2.cancer-pku.cn/index](http://gepia2.cancer-pku.cn/#index)), an interactive web server for analysing The Cancer Genome Atlas (TCGA) data, was used to assess *L1CAM* expression in the Stomach Adenocarcinoma (STAD) dataset for normal versus tumor samples. The correlation analysis module in GEPIA2 was utilized to assess the association between *L1CAM* and various integrins. Pearson's correlation test was applied to calculate the correlation coefficient (R) and mRNA expression values were normalized to GAPDH. The Tumor Immune Estimation Resource 2.0 (TIMER2.0) database (<http://timer.cistrome.org/>) was used to show *L1CAM* expression across cancers compared to normal tissues. The Human Protein Atlas (HPA) (<https://www.proteinatlas.org/>) was used to assess *L1CAM* expression across various cell lines in GC. To explore potential molecular interactions between L1CAM and other molecules, such as integrins, the Search Tool for the Retrieval of Interacting Genes/Proteins (STRING) (<https://string-db.org/>) was used.

**EV treatment**

For performing various in vitro functional assays and in vivo xenograft experiments, HFE145 and AGS cells, respectively, were treated with 20 µg of EVs isolated from uninfected or infected AGS cells, or from infected/uninfected empty vector- and L1CAM-stable AGS cells, for 24 h.

**Immunofluorescence/confocal microscopy**

0.2×10^6^ cells were plated on coverslips in 6-well plates. At the end of the experiment, cells were fixed with 4% PFA for 15 min at RT. Cells were then washed with PBS and permeabilized with Triton X-100 in PBS. Blocking was done using 5% bovine serum albumin (BSA) followed by overnight incubation with primary antibodies at 4 °C. The next day, cells were washed with PBST (0.1% tween20 in 1X PBS) and incubated with secondary antibodies in the dark. Lastly, cells were stained with DAPI and mounted with Fluoromount-G.

For human biopsy samples and xenograft tissue samples, 5 µm-thick tissue sections were sliced using a cryostat. Staining was performed in a similar manner as described above. Cell or tissue images were captured using a confocal or fluorescence microscope, respectively.

**Supplementary Tables**

**Table S1: List of reagents**

| **Cell line/Bacterial strain/Reagent** | **Manufacturer** | **Cat. number** |
| --- | --- | --- |
| AGS | ATCC | CRL-1739 |
| *Hp* 26695 | ATCC | 700392 |
| RPMI-1640 | HiMedia | AL028A |
| FBS | Invitrogen | 10270106 |
| Trypsin-EDTA solution | HiMedia | TCL007-500ML |
| Total exosome isolation | Invitrogen | 4478359 |
| Amicon® Ultra Centrifugal Filter, 100 kDa MWCO | Merck Millipore | UFC910096 |
| Bovine serum albumin (BSA) | HiMedia | GRM3151-100G |
| DAPI | Invitrogen | D3571 |
| GW280264X | Medchem express | HY-115670 |
| Fluoromount-G | SouthernBiotech | 0100-01 |
| RIPA buffer | HiMedia | TCL131 |
| PBS | HiMedia | ML023-500ML |
| Protease inhibitor cocktail | HiMedia | ML051-1ML |
| Prestained ladder | HiMedia | MBT092 |
| Polyvinylidene difluoride membranes | Merck Millipore | IPVH00010 |
| 5x loading buffer | HiMedia | ML021-6ML |
| Super Signal West Femto Chemiluminescent Substrate kit | Thermo Fisher Scientific | 34095 |
| Trypticase soy agar plates | Becton, Dickinson and Company; BD | 221261 |
| Brucella broth | BD | 211088 |
| Paraformaldehyde | Sigma-Aldrich | P6148 |
| 300 mesh copper grids | Electron Microscopy Sciences; EMS | CF300-Cu-50 |
| Uranyl acetate | EMS | 22405 |
| *phL1A*-pcDNA3 | Addgene | 12307 |
| control siRNA | Santa Cruz Biotechnology | sc-37007 |
| *NCAM-L1* siRNA | Santa Cruz Biotechnology | sc-43172 |
| G418 | Sigma-Aldrich | G8168 |
| Lipofectamine 3000 | Invitrogen | L3000001 |
| crystal violet solution | HiMedia | S012 |

**Table S2: List of antibodies**

| **Antibodies** | **Manufacturer (Cat. Number)** | **Application (Working dilution)** |
| --- | --- | --- |
| GAPDH | Abgenex (10-10011) | Western blotting (1:10000) |
| ALIX | Santa Cruz Biotechnology (sc-53540) | Western blotting (1:200) |
| HSPA8 | Cell Signalling Technology (1:1000) | Western blotting (1:1000) |
| GM130 | Novus Biologicals (NBP2-53420) | Western blotting (1:1000) |
| L1CAM | Abcam (ab24345) | Western blotting & IF (1:1000) |
| E-cadherin | Cell Signalling Technology (3195) | Western blotting (1:1000) |
| N-cadherin | Cell Signalling Technology (13116) | Western blotting (1:1000) |
| Snail | Cell Signalling Technology (3879) | Western blotting (1:1000) |
| Twist | Santa Cruz Biotechnology (sc-81417) | Western blotting (1:200) |
| Integrin αV | ABclonal Technology (A2091) | Western blotting (1:2000) & IF (1:100) |
| Integrin α5 | ABclonal Technology (A19069) | Western blotting (1:1000) |
| Integrin β1 | ABclonal Technology (A23497) | Western blotting (1:10000) & IF (1:400) |
| Integrin β3 | Santa Cruz Biotechnology (sc-46655) | Western blotting (1:200) |
| ADAM10 | ABclonal Technology (A25167) | Western blotting (1:1000) |
| ADAM17 | ABclonal Technology (A0821) | Western blotting (1:1000) |
| Anti-mouse | Cell Signaling Technology (7076S) | Western blotting (1:2000) |
| Anti-rabbit | Cell Signaling Technology (7074S) | Western blotting (1:2000) |
| EpCAM | Abcam (ab71916) | IF (1:160) |
| Ki67 | Abcam (ab15580) | IF (0.5µg/ml) |
| Alexa Fluor 488 | Invitrogen (A11055) | IF (1:2000) |
| Alexa Fluor 594 | Invitrogen (A21201) | IF (1:2000) |
