# Supplementary material for: Loss of L1CAM from gastric epithelial cells and extracellular vesicles promotes Helicobacter pylori-driven cancer progression and invasiveness
Source: Cell Commun Signal. 2026 Jul 25;24:484. doi: 10.1186/s12964-026-03087-5 (PMC13560308; doi:10.1186/s12964-026-03087-5)
Supplement: Supplementary file 2 [file 12964_2026_3087_MOESM2_ESM.docx]

**Supplementary figures and legends**

**Supplementary Fig. 1.** *L1CAM e*xpression is significantly reduced in TCGA-STAD and various GC cells. (A) TIMER2.0 analysis reveals cancer-type specific variability in *L1CAM* expression, including STAD (highlighted in yellow box). (B) Immunoblots showing L1CAM status in HFE145, AGS and MKN45 cells. Bar graph (n = 3) shows L1CAM fold change relative to GAPDH. GAPDH is the loading control. (C) *L1CAM* expression analysis across various GC cell types using HPA, including AGS and MKN45 (highlighted in yellow boxes). Statistical significance is determined by one-way ANOVA followed by Tukey’s post hoc analysis. Data are presented as mean ± SEM. *, *P*< 0.05; **, *P*< 0.01; ***, *P*< 0.001.

**Supplementary Fig. 2.** L1CAM is significantly reduced in GECs following *Hp* infection. (A) DLS analysis of EVs isolated from *Hp*-infected/uninfected GECs. Western blot results showing L1CAM expression in WCLs and EVs obtained from (B) HFE145 and (C) MKN45. Bar graph (n = 3) (yellow bar graphs for HFE145 and pink bars graphs for MKN45 cells) represents the fold change in L1CAM relative to GAPDH. (D) Western blotting of WCLs and EVs derived from AGS cells infected with 26695 *cag* PAI (+) or 8-1 *cag* PAI (-) strain of *Hp*. Graphs (n = 3) represent fold change in L1CAM relative to GAPDH in WCL or EV. GAPDH is used as the loading control. Statistical significance is determined by one-way ANOVA followed by Tukey’s post hoc analysis. Data are presented as mean ± SEM.*, *P*< 0.05; **, *P*< 0.01; ***, *P*< 0.001; ****, *P*< 0.0001; ns, non-significant.

**Supplementary Fig. 3.** *Hp* infection-dependent L1CAM downregulation promotes EMT. (A) Immunoblots showing the status of EMT markers in uninfected or infected AGS cells. The bar graph (n = 3) represents relative fold change of N-cadherin, Snail and Twist1 with respect to GAPDH. (B) Immunoblot analysis of EMT markers in empty vector and *L1CAM* siRNA-transfected AGS cells with or without infection (n = 3). GAPDH is the loading control. Statistical significance is determined by Student’s t-test and one-way ANOVA followed by Tukey’s post hoc analysis. Data are presented as mean ± SEM. *, *P*< 0.05; **, *P*< 0.01; ***, *P*< 0.001; ****, *P*< 0.0001.

**Supplementary Fig. 4.** L1CAM loss in *Hp* infection is associated with reduced integrin αV and β1 levels in GC. (A) Correlation analysis of *L1CAM* with *ITGA5* and *ITGB3* using GEPIA2. (B) Bar graphs (n = 3) showing the relative fold change in MFI of integrin αV and integrin β1 in uninfected and *Hp*-infected empty vector and *L1CAM* siRNA-transfected AGS cells, corresponding to the data presented in Fig. 6A. (C) Accompanying bar graphs (n = 3) represent the relative fold change in MFI of integrin αV and integrin β1 in *Hp*-positive GC biopsies and *Hp*-negative peritumoral controls, corresponding to the immunofluorescence data shown in Fig. 6B. (D) Graphs (n = 5) represent relative fold change in MFI of integrin αV and integrin β1 in EV-treated xenograft tumors derived from empty vector and L1CAM-stable cells, with/without *Hp* infection, corresponding to immunofluorescence data shown in Fig. 6C. Statistical significance is determined by one-way ANOVA followed by Tukey’s post hoc analysis and Student’s t-test. Data are presented as mean ± SEM. *, *P*< 0.05; **, *P*< 0.01; ***, *P*< 0.001; ****, *P*< 0.0001; ns, non-significant; MFI, mean fluorescence intensity.

**Supplementary Fig. 5.** L1CAM fragments appear in EVs. (A) Representative immunoblot showing L1CAM cleavage fragments in EVs derived from AGS cells with or without *Hp* infection and in the presence or absence of ADAM10/17 inhibitor.
